# Off-resonance rotating-frame relaxation dispersion experiment for ^13^C in aromatic side chains using L-optimized TROSY-selection

**DOI:** 10.1007/s10858-014-9826-2

**Published:** 2014-04-05

**Authors:** Ulrich Weininger, Ulrika Brath, Kristofer Modig, Kaare Teilum, Mikael Akke

**Affiliations:** 1 Department of Biophysical Chemistry, Center for Molecular Protein Science, Lund University, P.O. Box 124, 22100 Lund, Sweden; 2 Present Address: Department of Chemistry and Molecular Biology, University of Gothenburg, 41296 Göteborg, Sweden; 3 Department of Biology, University of Copenhagen, Ole Maaløes Vej 5, 2200 Copenhagen, Denmark

**Keywords:** Conformational exchange, Strong coupling, Aromatic ring flip, Spin-lock

## Abstract

Protein dynamics on the microsecond–millisecond time scales often play a critical role in biological function. NMR relaxation dispersion experiments are powerful approaches for investigating biologically relevant dynamics with site-specific resolution, as shown by a growing number of publications on enzyme catalysis, protein folding, ligand binding, and allostery. To date, the majority of studies has probed the backbone amides or side-chain methyl groups, while experiments targeting other sites have been used more sparingly. Aromatic side chains are useful probes of protein dynamics, because they are over-represented in protein binding interfaces, have important catalytic roles in enzymes, and form a sizable part of the protein interior. Here we present an off-resonance *R*_1ρ_ experiment for measuring microsecond to millisecond conformational exchange of aromatic side chains in selectively ^13^C labeled proteins by means of longitudinal- and transverse-relaxation optimization. Using selective excitation and inversion of the narrow component of the ^13^C doublet, the experiment achieves significant sensitivity enhancement in terms of both signal intensity and the fractional contribution from exchange to transverse relaxation; additional signal enhancement is achieved by optimizing the longitudinal relaxation recovery of the covalently attached ^1^H spins. We validated the L-TROSY-selected *R*_1ρ_ experiment by measuring exchange parameters for Y23 in bovine pancreatic trypsin inhibitor at a temperature of 328 K, where the ring flip is in the fast exchange regime with a mean waiting time between flips of 320 μs. The determined chemical shift difference matches perfectly with that measured from the NMR spectrum at lower temperatures, where separate peaks are observed for the two sites. We further show that potentially complicating effects of strong scalar coupling between protons (Weininger et al. in J Phys Chem B 117: 9241–9247, 2013b) can be accounted for using a simple expression, and provide recommendations for data acquisition when the studied system exhibits this behavior. The present method extends the repertoire of relaxation methods tailored for aromatic side chains by enabling studies of faster processes and improved control over artifacts due to strong coupling.

Conformational fluctuations in proteins on the microsecond to millisecond time scales are often linked to functional processes (Mittermaier and Kay 2006). Transitions between different conformations that lead to modulation of NMR parameters, such as the chemical shift (Gutowsky and Saika 1953) or residual dipolar couplings (Igumenova et al. 2007; Vallurupalli et al. 2007), result in exchange contributions to the transverse relaxation rate, which can be probed by NMR relaxation dispersion methods to yield unique information on the structures, thermodynamics and dynamics of the underlying process (Palmer et al. 2001; Akke 2002). Experiments have been designed to probe conformational exchange at specific sites in proteins, including the backbone (Akke and Palmer 1996; Ishima et al. 1998, 2004; Loria et al. 1999a, b; Hill et al. 2000; Mulder and Akke 2003; Lundström and Akke 2005a, b; Igumenova and Palmer 2006; Lundström et al. 2009a) and side-chain aliphatic (Lundström et al. 2007b, 2009b; Hansen et al. 2012), methyl (Ishima et al. 1999; Mulder et al. 2002; Brath et al. 2006; Lundström et al. 2007b; Weininger et al. 2012b, 2013a), carbonyl/carboxyl (Paquin et al. 2008; Hansen and Kay 2011) and recently also aromatic groups (Teilum et al. 2006; Weininger et al. 2012c, 2013b).

Aromatic residues serve multiple functions in proteins. They commonly occur in protein binding interfaces, where they contribute a significant part of the binding free energy (Bogan and Thorn 1998; Lo Conte et al. 1999; Birtalan et al. 2010). Furthermore, His and Tyr play critical roles in enzyme catalytic mechanisms (Bartlett et al. 2002). Aromatic side chains contribute some 25 % of the protein interior volume, typically appearing in pairs or clusters where they form specific aromatic–aromatic interactions (Burley and Petsko 1985). Despite the generally tight packing of protein side chains, Phe and Tyr residues undergo frequent 180° rotations (‘ring flips’) of the *χ* ^2^ dihedral angle. For ring flips to occur, the available volume surrounding the ring must increase transiently (Wagner 1980; Karplus and McCammon 1981; Li et al. 1999). Therefore, aromatic residues are useful probes of the dynamics of the hydrophobic core. Indeed, studies of aromatic ring dynamics have a very long history in NMR, dating back to the 1970s when aromatic ring flips were first observed in proteins (Wüthrich and Wagner 1975; Campbell et al. 1975; Hull and Sykes 1975; Wagner et al. 1976) and has recently seen a renaissance (Skalicky et al. 2001; Sathyamoorthy et al. 2013; Weininger et al. 2013b; Kasinath et al. 2013).

Previous studies of ring-flip rates have largely been based on proton-detected lineshape analysis and various types of exchange spectroscopy (Li et al. 1999; Skalicky et al. 2001; Hattori et al. 2004; Rao and Bhuyan 2007). Recent developments have made it possible to study conformational dynamics of aromatic side-chains using heteronuclear relaxation rate measurements: site-specific ^13^C labeling of proteins using 1-^13^C_1_-glucose or 2-^13^C_1_-glucose produces samples with isolated ^13^C spins, thereby eliminating unwanted relaxation pathways and coherent magnetization transfer via one-bond couplings (Teilum et al. 2006; Lundström et al. 2007a) and enabling the first studies of conformational exchange of aromatic rings using *R*_1ρ_ relaxation dispersion (Teilum et al. 2006). Alternative labeling strategies using 4-^13^C_1_-erythrose (Kasinath et al. 2013) or α-ketoacid precursors (Lichtenecker et al. 2013) in combination with deuteration lead to protein samples with higher isotope enrichment level and higher degree of isolation of the ^1^H–^13^C spin pairs in the aromatic rings of Phe and Tyr.

We recently introduced a suite of ^13^C longitudinal and transverse relaxation-optimized (L-TROSY) based relaxation experiments, which offer improvements in signal-to-noise per unit time of at least 10–35 % (Weininger et al. 2012a). By measuring the relaxation of the narrow multiplet component, TROSY-type experiments significantly improve the relatively low sensitivity of aromatic ^13^C spins towards exchange contributions to *R*_2_ caused by their inherently fast transverse relaxation (approximately a factor of 4 greater than for a backbone ^15^N spin) (Weininger et al. 2012c). We have shown that anomalous, ‘upside-down’ dispersion profiles recorded using the L-TROSY CPMG experiment are caused by strong ^1^H-^1^H couplings (Weininger et al. 2013b). In favorable cases, the anomalous dispersion profiles can be interpreted to determine slow ring-flip rates, even though the observed nuclei have near-degenerate chemical shifts in the two positions (Weininger et al. 2013b). These results clearly show that renewed interest in aromatic spin relaxation (Skalicky et al. 2001; Teilum et al. 2006; Boyer and Lee 2008; Weininger et al. 2012a, c; Kasinath et al. 2013) is rewarded by new insights into protein dynamics. To further expand the repertoire of spin relaxation experiments targeting aromatic rings, we introduce the ^13^C L-TROSY-selected *R*_1ρ_ experiment, which complements the corresponding CPMG-type experiment (Weininger et al. 2012c) by enabling studies of faster processes, as well as providing improved control over the effect of strong ^1^H-^1^H coupling on the acquired dispersion profiles.

We implemented L-TROSY in the context of the off-resonance *R*_1ρ_ relaxation dispersion experiment (Akke and Palmer 1996; Zinn-Justin et al. 1997; Mulder et al. 1998; Evenäs et al. 2001), in order to study faster exchange processes involving aromatic rings. The pulse sequence (Fig. 1) uses the same general framework as the L-TROSY versions of the *R*_1_, *R*_2_, *NOE* (Weininger et al. 2012a) and CPMG dispersion experiments (Weininger et al. 2012c), where L-optimization is achieved by maintaining the water and aliphatic magnetizations along the +*z*-axis; see (Weininger et al. 2012a) for details. The present *R*_1ρ_ relaxation experiment encompasses spin-state selection so as to spin-lock the narrow C_x_H^α^ component of the ^13^C doublet, as described previously for the analogous TROSY-selected *R*_1ρ_ experiment developed for ^15^N spins (Igumenova and Palmer 2006). The total relaxation period *T* is divided into two segments of equal length, interspersed by a ^1^H decoupling element. In short, C_z_H^α^ magnetization is initially generated at point A (Fig. 1) using a heteronuclear S^3^E element (Meissner et al. 1997). The ^13^C magnetization is then aligned along the effective field axis using an adiabatic *B*_1_ profile, spin-locked for a period *T*/2, and subsequently realigned along the *z*-axis at point B using a time-reversed adiabatic profile. ^1^H inversion is achieved using an S^3^CT element (Sorensen et al. 1997) between points B and C. Finally, the second half of the spin-lock period is executed identically to the first half, returning C_z_H^α^ magnetization at point D. The present approach differs from that employed in the corresponding CPMG experiments (Loria et al. 1999b; Vallurupalli et al. 2007; Weininger et al. 2012c), which use an S^3^CT element as the sole selection filter. The TROSY-selected off-resonance *R*_1ρ_ experiment uses selective excitation, as well as selective inversion, of the slowly relaxing doublet component to minimize unwanted cross-relaxation effects (Igumenova and Palmer 2006), and also maintains a well-defined tilt-angle throughout the entire relaxation period, which further avoids artifacts (Korzhnev et al. 2002; Massi et al. 2004).

**Fig. 1:**
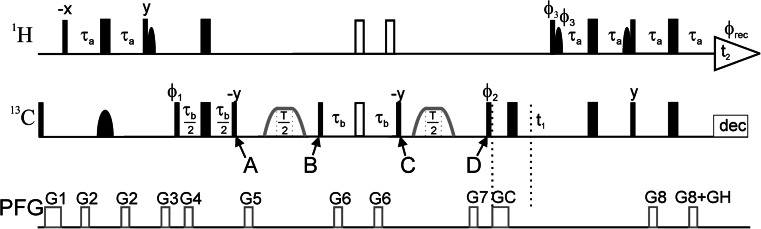
Pulse sequence of the L-TROSY-*R*_1ρ_ relaxation dispersion experiment for measuring conformational exchange of aromatic side chains in specifically ^13^C labeled proteins. All pulses are applied along the *x axis* unless otherwise indicated. *Narrow* (*wide*) *solid bars* indicate rectangular high-power 90° (180°) pulses. *Open wide bars* indicate composite 180° pulses. The *continuous*-*wave* spin-lock relaxation periods T/2 and their flanking 4 ms tan/tanh adiabatic profiles are outlined in *gray* between points marked *A* and *B*, and *C* and D. The adiabatic sweep is initiated 25 kHz downfield or upfield of the spin-lock frequency. *Solid semi*-*ellipses* represent shaped pulses. *Narrow semi*-*ellipses* on ^1^H are 90° EBURP2 (Geen and Freeman 1991) shapes centered at 1.9 ppm with a bandwidth of 6.6 ppm. The *wide semi*-*ellipse* on ^13^C represents a 180° REBURP (Geen and Freeman 1991) pulse with a bandwidth of 40 ppm. ^13^C is decoupled during acquisition using GARP (Shaka et al. 1985). The delays τ_a_ and τ_b_ are set to 1.5 and 1.4 ms, respectively. The magnetizations from water and aliphatic ^1^H spins are aligned along the +*z* axis whenever possible, including the spin-lock periods. The phase cycle is: ϕ_1_ = 4(135°), 4(−45°), ϕ_2_ = (*y, x,* −*y,* −*x*), ϕ_3_ = (−*y*), ϕ_rec_ = (*x,* −*y,* −*x, y,* −*x, y, x,* −*y*). Pulsed field gradients G1–8 are employed to suppress unwanted coherences and artifacts, while GC and GH are encoding and decoding gradients, respectively, for echo/anti-echo coherence selection, obtained by inverting the signs of ϕ_3_, GC and the even-numbered phases of the receiver. Gradient durations (in ms) and power levels (G/cm) are set to: G1 = (1.0, 10), G2 = (0.5, 8), G3 = (0.5, −20), G4 = (0.5, 6), G5 = (0.5, −10), G6 = (0.5, 12), G7 = (0.5, −40), G8 = (0.5, 18), GC = (1.0, 54), GH (0.5, 27.018). For every second t_1_ increment, ϕ_2_ and the receiver were incremented

The TROSY effect for aromatic ^13^C spins is expected to be near-optimal at a static magnetic field strength of *B*_0_ = 14.1 T, as calculated based on the chemical shielding tensor for benzene (Veeman 1984) (*σ*_11_ = 225 ppm, *σ*_22_ = 149 ppm, *σ*_33_ = 15 ppm) and a C–H bond length of 1.08 Å. For reference, the estimated relaxation rate of the C_x_H^α^ magnetization for an isotropically tumbling protein with an order parameter of *S* ^2^ = 0.85, varies between 8 and 30 s^−1^ for rotational correlation times in the range of 5–20 ns.

To validate the ^13^C L-TROSY-selected *R*_1ρ_ pulse sequence, we measured the ring-flip rate of Y23 in an 8 mM sample of natural abundance bovine pancreatic trypsin inhibitor (BPTI) (Wagner et al. 1976), dissolved in water pH 7.1, at a temperature of 55 °C and a static magnetic field strength of 11.7 T. Under these conditions, a single peak is observed for the *δ* spins, as well as the *ε* spins, and the ring flip is too fast to be determined accurately by CPMG relaxation dispersion (see Fig. 2). The advantage of benchmarking the performance of the *R*_1ρ_ pulse sequence against aromatic ring flips in the fast exchange regime is that one can extract the chemical shift difference (Δ*δ*) from the product *ϕ*_ex_ = (Δ*δ*)^2^ *p*_1_ *p*_2_, since in this case the populations are known a priori (*p*_1_ = *p*_2_ = 0.5). Consequently, the value of Δ*δ* determined from the *R*_1ρ_ experiment can be directly compared to that determined from a spectrum recorded at lower temperature where the ring flip is in the slow exchange regime. Miloushev and Palmer have described a closed analytical formula for the specific case of symmetric two-state exchange in the fast exchange regime, expressing *R*_1ρ_ as a function of the longitudinal relaxation rate (*R*_1_), the exchange-free transverse relaxation rate (*R*_2,0_), the exchange rate (*k*_ex_), the chemical shift difference between the exchanging sites (Δ*δ*), and the effective field strengths of the two exchanging sites (*ω*_e_ = (*ω*_1_^2^ + *ΔΩ* ^2^)^1/2^), which is the vector sum of the *B*_1_ field strength (*ω*_1_) and the off-resonance frequencies (*ΔΩ*), both of which are variables under experimental control (Miloushev and Palmer 2005).

**Fig. 2:**
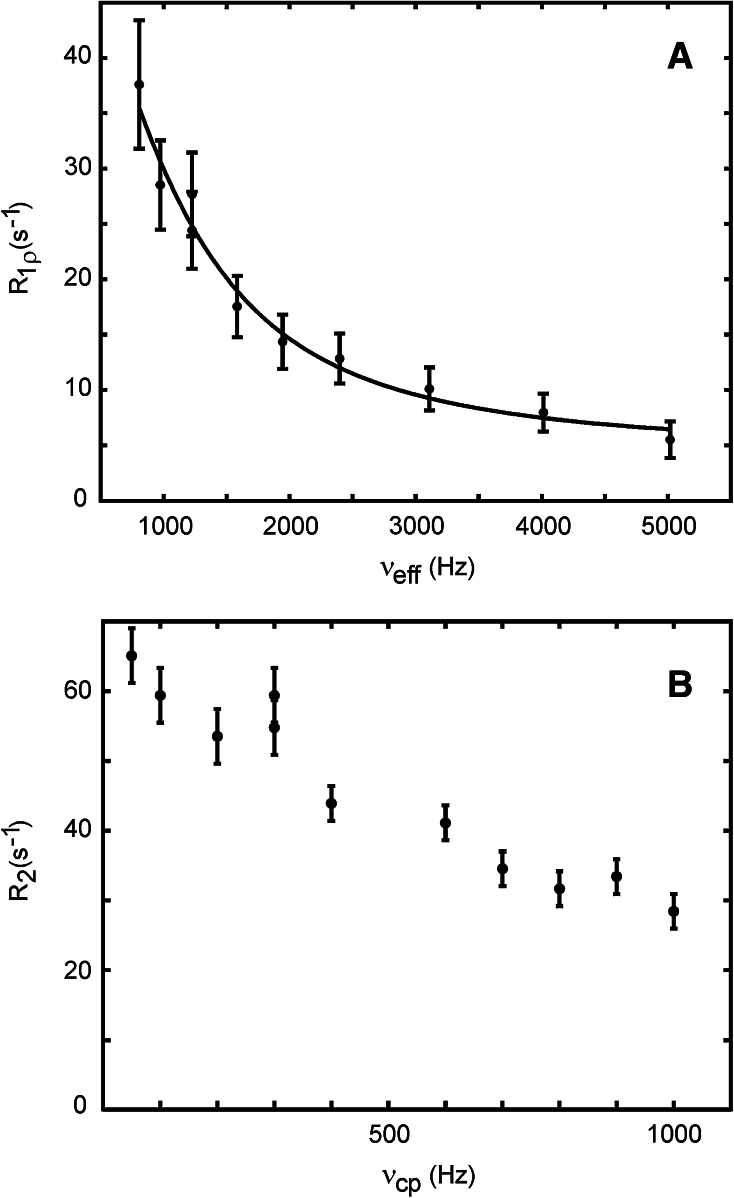
^13^C aromatic L-TROSY-selected *R*_1ρ_ (**a**) and CPMG (**b**) relaxation dispersion data for Y23 ^13^C^ε^ acquired on an 8 mM sample of natural abundance BPTI in water pH 7.1 at 55 °C and a static magnetic field strength of 11.7 T. The *R*_1ρ_ experiment was performed with the carrier placed on resonance with respect to the exchange-averaged ^13^C signal. Data were fitted using the equation for symmetric exchange (Miloushev and Palmer 2005) and fixed populations, *p*_1_ = *p*_2_ = 0.5. The fitted parameters are *k*_ex_ = (6.2 ± 2.1) × 10^3^ s^−1^ and Δ*δ* = 1.43 ± 0.09 ppm

Using the Miloushev-Palmer equation for the equal populations condition, we obtain a chemical shift difference for ^13^C^*ε*^ of Y23 of Δ*δ* = 1.43 ± 0.09 ppm and *k*_ex_ = (6.2 ± 2.1) × 10^3^ s^−1^. The chemical shift difference is in very good agreement with that measured (Δ*δ* = 1.50 ppm) at 5 and 15 °C, where Y23 is in slow exchange and separate signals from each side of the ring can be seen directly in the spectra; since the shift difference is virtually constant between 5 and 15 °C, we take this value to hold also at 55 °C. The stated errors correspond to one standard deviation, obtained by repeating the non-linear least square fit 1,000 times in a Monte-Carlo fashion (Press et al. 1986).

We have previously shown that aromatic ^13^C L-TROSY CPMG relaxation dispersion experiments are affected by strong ^3^ *J*_HH_ couplings between the proton directly attached to the ^13^C of interest and its vicinal ^12^C-attached neighbor (Weininger et al. 2013b). Below, we outline the effect of strong ^3^ *J*_HH_ couplings in the context of the ^13^C L-TROSY-selected *R*_1ρ_ experiment, starting with a brief summary of the general conclusions presented previously. Strong ^3^ *J*_HH_ coupling leads to anomalous dispersion profiles, caused by modulation of the strong-coupling parameter *ψ* (defined by tan(2*ψ*) = ^3^ *J*_HH_/Δ*ν*) as a function of the ^13^C refocusing frequency (Weininger et al. 2013b), as exemplified in Fig. 3a, c. Phe, Tyr and the 6-ring moiety of Trp can be affected by strong couplings in principle, while His and the 5-ring moiety of Trp cannot, since the neighboring proton in each of the latter cases is attached to nitrogen and therefore resonates at a frequency far away from that of the ^13^C-attached proton, i.e. Δ*ν*_HH_ ≫ ^3^ *J*_HH_. The frequency difference between the two protons is given by Δ*ν* = Δ*ν*_HH_ + ^1^ *J*_CH,eff_, where Δ*ν*_HH_ is the difference in resonance frequency between the ^12^C-coupled proton and the central (decoupled) line of the ^13^C-coupled proton (e.g. the *δ* and *ε* protons on each side of the ring in Phe and Tyr), and ^1^ *J*_CH,eff_ is the effective scalar coupling constant of the ^1^H–^13^C pair under the given ^13^C decoupling conditions of the CPMG or spin-lock sequence. The ^1^H-^1^H scalar coupling constant is essentially invariable in Phe and Tyr aromatic rings, ^3^ *J*_HH_ ≈ 7–8 Hz (Laatikainen et al. 1995), so that strong scalar coupling applies if Δ*ν* < 2.5·^3^ *J*_HH_. To determine whether the strong-coupling condition applies on either side of the ring, one thus needs information on Δ*ν*_HH_, which can be measured directly from the spectrum only if the ring-flip rate is slow enough that separate cross-peaks are observed for the two symmetric sites. In the case that only a single resonance is observed for each of the *δ* and *ε* protons (with ^13^C decoupling), it is still possible to determine whether strong coupling applies to one or both sides of the ring, based on the observed chemical shift difference between the two protons, Δ*ν*_HH,obs_ = (Δ*ν*_HH,1_ + Δ*ν*_HH,2_)/2 = (*ν*_*δ*1_ − *ν*_*ε*1_ + *ν*_*δ*2_ − *ν*_*ε*2_)/2, and the criterion Δ*ν*_HH_ < 2.5·^3^ *J*_HH_ (Weininger et al. 2013b); subscripts 1 and 2 denote the two sides of the ring. In the case of strong coupling on only one side of the ring, an anomalous dispersion profile implies that the ring-flip rate is quite slow (<200 s^−1^), despite the appearance of a single cross-peak, since faster ring-flip rates effectively quench the strong coupling by exchange averaging (Weininger et al. 2013b).

**Fig. 3:**
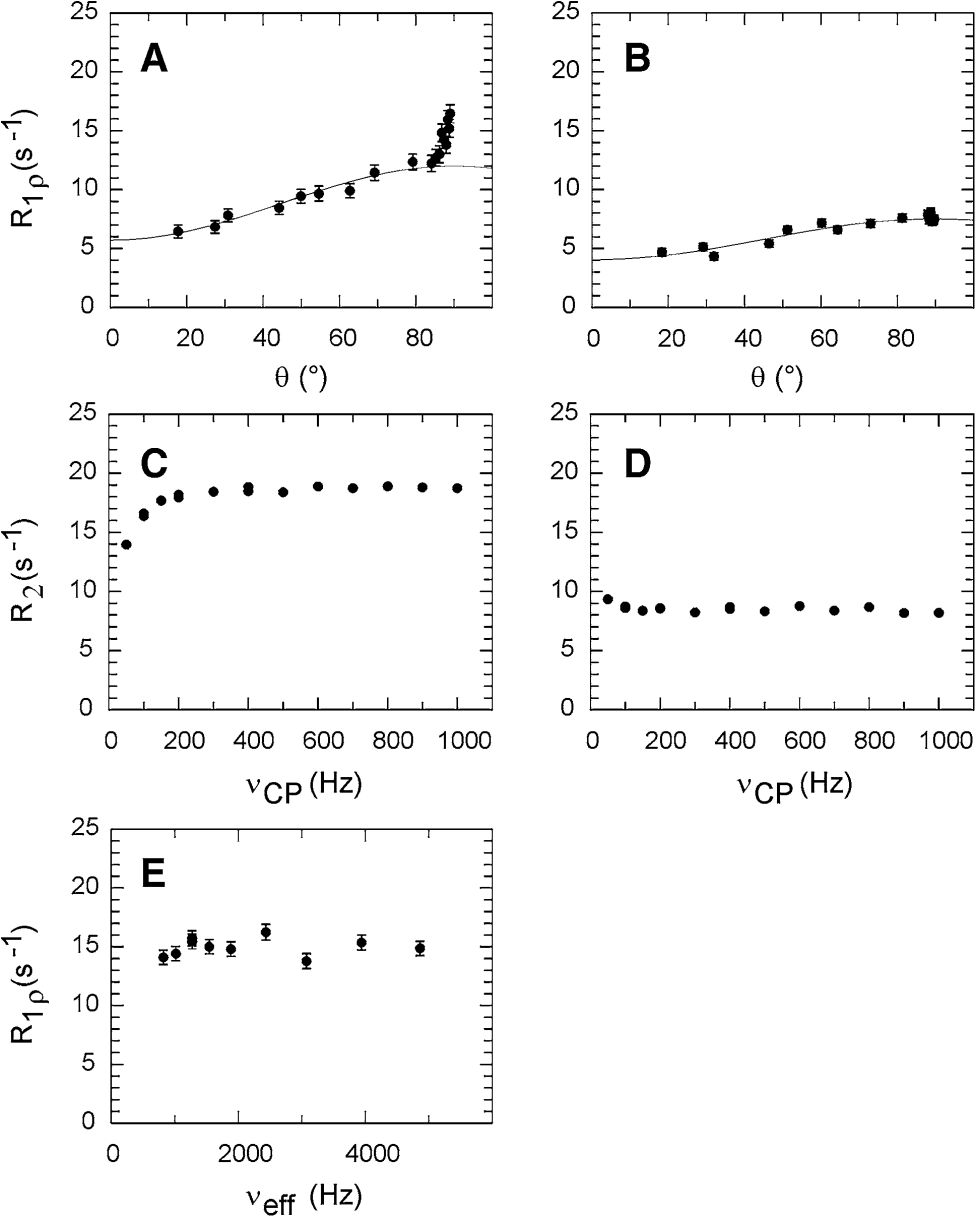
Aromatic ^13^C relaxation dispersion profiles of SlyD F79δ (**a**, **c**, **e**) and Y92δ (**b**, **d**). Neither of these residues are significantly influenced by exchange. H^δ^ and H^ε^ are strongly coupled in F79, but weakly coupled in Y92. **a**, **b** *R*_1ρ_ relaxation dispersion shown as a function of the tilt angle *θ*. Data were fitted using the exchange-free expression *R*_1ρ_ = *R*_1_cos^2^(θ) + *R*_2_sin^2^(θ) using *θ* < 80°. **c**, **d** CPMG relaxation dispersion profiles. **e** On-resonance (*θ* = 90°) *R*_1ρ_ relaxation dispersion of F79δ. Data were acquired on a 1 mM sample of 1-^13^C_1_ glucose-labeled SlyD in 20 mM HEPES, pH 7.4 at 25 °C and a static magnetic field strength of 11.7 T

To investigate the effect of strong couplings on *R*_1ρ_ relaxation dispersions, we acquired off-resonance *R*_1ρ_ data at different tilt angles (corresponding to different ^13^C refocusing frequencies) on a 1 mM sample of 1-^13^C_1_ glucose-labeled SlyD from *Thermus thermophilus* (Löw et al. 2010), dissolved in 20 mM HEPES pH 7.4, at a temperature of 25 °C and a static magnetic field strength of 11.7 T. In SlyD, residue F79 exhibits strong ^3^ *J*_HH_ coupling on both sides of the ring (i.e., Δ*ν*_HH,1_, Δ*ν*_HH,2_ < 2.5·^3^ *J*_HH_), while all other aromatic residues are weakly coupled. (The *δ* and *ε* protons of Y23 in BPTI are weakly coupled on both sides of the ring; data not shown). As seen from Fig. 3a, strong coupling causes an anomalous increase in *R*_1ρ_ relaxation rates of F79 for tilt angles *θ* > 80°. This is a general result that is fully explained by the decoupling efficiency of the continuous-wave (cw) spin-lock field. The ^13^C cw spin-lock field employed in the *R*_1ρ_ experiment scales the splitting of the ^13^C-coupled proton in a predictable manner according to ^1^ *J*_CH,eff_ = ^1^ *J*_CH_ *ΔΩ*/(*ω*_1_^2^ + *ΔΩ* ^2^)^1/2^ = ^1^ *J*_CH_ cos*θ* (Shaka and Keeler 1987). We verified the expected dependence by measuring the residual splitting in the ^1^H dimension of a ^1^H–^13^C HSQC acquired with off-resonance cw ^13^C decoupling during acquisition (Fig. 4). In the case Δ*ν*_HH_ ≈ 0, strong coupling thus arises for ^1^ *J*_CH,eff_ < 2.5·^3^ *J*_HH_, which translates to tilt angles *θ* > 80° (Fig. 4). As evident from Fig. 3a, it is quite straightforward in this case to identify the effect of strong coupling in the *R*_1ρ_ experiment and subsequently exclude data points at *θ* > 80° from further analysis. In cases where Δ*ν*_HH_ ≠ 0, the strong coupling scenario might be reached at intermediate values of *θ*, but typically affects only a single point on the relaxation dispersion curve (data not shown), which can be omitted provided that it can be reliably identified. Alternatively, the effects of strong-coupling artifacts on individual data points can be mitigated by acquiring *R*_1ρ_ dispersion data using a fixed tilt angle but variable effective field strength. In this case strong coupling increases the measured *R*_1ρ_ rates by a constant value that does not influence the final dispersion profile, as exemplified by Fig. 3e. This approach is advantageously performed on-resonance for cases where Δ*ν*_HH_ ≠ 0, which yields the greatest exchange contributions to the measured *R*_1ρ_ rates, while a value of *θ* ≈ 80° should be near-optimal for Δ*ν*_HH_ ≈ 0. Thus, the *R*_1ρ_ experiment offers a higher level of control over the effects of strong ^3^ *J*_HH_ coupling on the dispersion profile than what is possible in CPMG experiments, where decoupling generates sidebands that modulate the splitting of the ^1^H resonances in a manner that cannot be predicted quantitatively without numerical simulations (Weininger et al. 2013b; Eden et al. 1996).

**Fig. 4:**
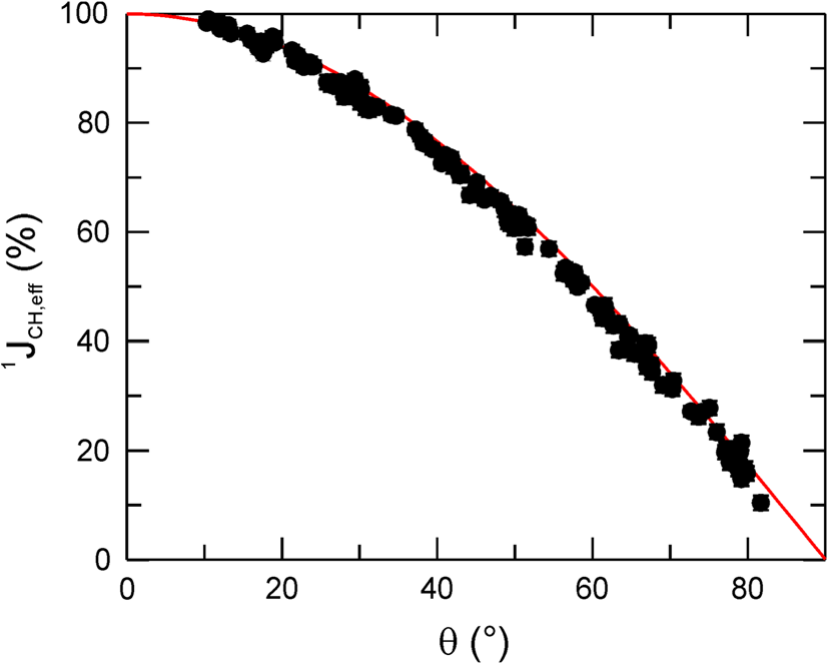
The relative effective ^1^H-^13^C scalar coupling constant, ^1^ *J*_CH,eff_ (%), plotted as a function of the tilt angle *θ*. The expected dependence ^1^ *J*_CH,eff_ = ^1^ *J*_CH_ cos*θ* is indicated as a *solid*, *red line*. Data were acquired using cw ^13^C decoupling during the acquisition of aromatic ^1^H–^13^C-HSQC spectra on a 1 mM sample of 1-^13^C_1_ glucose-labeled SlyD in 20 mM HEPES, pH 7.4 at 25 °C, and a static magnetic field strength of 11.7 T

In conclusion, the ^13^C L-TROSY-selected *R*_1ρ_ pulse sequence enables accurate measurements of conformational exchange affecting aromatic rings. The effect of strong ^1^H-^1^H scalar couplings on the dispersion profile can be predicted reliably using a closed analytical expression describing the ^13^C decoupling efficiency during the spin-lock and prior knowledge of the ^1^H spectrum. The new pulse sequence complements previous experiments, such as the L-TROSY CPMG dispersion experiment, by extending the accessible range of exchange processes towards faster rates and by offering additional advantages intrinsic to rotating-frame experiments (Palmer and Massi 2006).
